# Glutaredoxins are essential for stress adaptation in the cyanobacterium *Synechocystis* sp. PCC 6803

**DOI:** 10.3389/fpls.2013.00428

**Published:** 2013-11-04

**Authors:** Ana M. Sánchez-Riego, Luis López-Maury, Francisco J. Florencio

**Affiliations:** Instituto de Bioquímica Vegetal y Fotosíntesis, Universidad de Sevilla-CSICSevilla, Spain

**Keywords:** glutaredoxin, stress, redox regulation, cyanobacteria, high light, heat shock, oxidative stress, metal resistance

## Abstract

Glutaredoxins are small redox proteins able to reduce disulfides and mixed disulfides between GSH and proteins. *Synechocystis* sp. PCC 6803 contains three genes coding for glutaredoxins: *ssr2061* (*grxA*) and *slr1562* (*grxB*) code for dithiolic glutaredoxins while *slr1846* (*grxC*) codes for a monothiolic glutaredoxin. We have analyzed the expression of these glutaredoxins in response to different stresses, such as high light, H_2_O_2_ and heat shock. Analysis of the mRNA levels showed that *grxA* is only induced by heat while *grxC* is repressed by heat shock and is induced by high light and H_2_O_2_. In contrast, *grxB* expression was maintained almost constant under all conditions. Analysis of GrxA and GrxC protein levels by western blot showed that GrxA increases in response to high light, heat or H_2_O_2_ while GrxC is only induced by high light and H_2_O_2_, in accordance with its mRNA levels. In addition, we have also generated mutants that have interrupted one, two, or three glutaredoxin genes. These mutants were viable and did not show any different phenotype from the WT under standard growth conditions. Nevertheless, analysis of these mutants under several stress conditions revealed that single *grxA* mutants grow slower after H_2_O_2_, heat and high light treatments, while mutants in *grxB* are indistinguishable from WT. *grxC* mutants were hypersensitive to treatments with H_2_O_2_, heat, high light and metals. A double *grxAgrxC* mutant was found to be even more sensitive to H_2_O_2_ than each corresponding single mutants. Surprisingly a mutation in *grxB* suppressed totally or partially the phenotypes of *grxA* and *grxC* mutants except the H_2_O_2_ sensitivity of the *grxC* mutant. This suggests that *grxA* and *grxC* participate in independent pathways while *grxA* and *grxB* participate in a common pathway for H_2_O_2_ resistance. The data presented here show that glutaredoxins are essential for stress adaptation in cyanobacteria, although their targets and mechanism of action remain unidentified.

## Introduction

Glutaredoxins are small redox proteins that were first discovered as alternatives to thioredoxin as electron donors for ribonucleotide reductase (Holmgren, 1976). Glutaredoxin structures are closely related to those of thioredoxins, the so-called thioredoxin fold, and they also catalyze disulfide reduction through a dithiol mechanism, as thioredoxins, though some glutaredoxins use a monothiol mechanism (Fernandes and Holmgren, 2004; Meyer et al., 2009). Furthermore, glutaredoxins are able to catalyze protein glutathionylation/deglutathionylation, a function initially ascribed for these enzymes (Rouhier et al., 2008; Meyer et al., 2009), but recent data suggest that thioredoxins could also contribute to deglutathionylating reaction in yeast and plants (Greetham et al., 2010; Bedhomme et al., 2012). Glutaredoxins generally use the GSH/glutathione reductase system for reduction, although some of them can also accept electrons directly from thioredoxin reductases or can be reduced by thioredoxins or other glutaredoxins (Zaffagnini et al., 2008; Couturier et al., 2009a; Meyer et al., 2009). Glutaredoxins can function as alternative electron donors for ribonucleotide reductase in *E. coli* (but not in yeast), in sulfate assimilation, as electron donors for 3′-phosphoadenosine 5′-phosphosulfate synthase or for methionine sulfoxide reductases (Meyer et al., 2009; Hanschmann et al., 2013; Lillig and Berndt, 2013; Toledano et al., 2013). Moreover, some glutaredoxins present peroxidase activity and/or are able to reduce some peroxiredoxins thus contributing to resistance to oxidative stress (Rouhier et al., 2001, 2002; Finkemeier et al., 2005; Hanschmann et al., 2010; Pedrajas et al., 2010). In addition, it has also been shown that some glutaredoxins are able to bind Fe-S clusters and are involved in some aspects of Fe-S cluster biogenesis and its regulation (Muhlenhoff et al., 2010; Rouhier, 2010; Couturier et al., 2011; Kumar et al., 2011; Li and Outten, 2012; Boutigny et al., 2013).

Six different classes of glutaredoxins have been described in photosynthetic organisms (Couturier et al., 2009a), where class I and II correspond to classical dithiolic and monothiolic glutaredoxins, respectively, that are present in all organisms. Class IV is restricted to photosynthetic eukaryotes and class III is specific to land plants. On the other hand class VI seems to be restricted to cyanobacteria and class V is only present in cyanobacteria and some proteobacteria (Benyamina et al., 2013). In photosynthetic eukaryotes the repertoire of glutaredoxin proteins is larger than in other organisms, which suggests that they could play critical roles regulating processes related to photosynthesis (Rouhier et al., 2008; Couturier et al., 2009a). The exact roles of glutaredoxins in photosynthetic organisms remain unclear mainly because several paralogs of each have been identified in many plant genomes and only a few mutants are available (Couturier et al., 2009a; Meyer et al., 2009, 2012). In *Arabidopsis* only a few mutants in glutaredoxin genes have been analyzed. GrxS14 and GrxS17 class II glutaredoxins have been shown to be essential for oxidative stress tolerance and heat tolerance, respectively, while class III glutaredoxins have been shown to be essential for flower development and pathogen resistance (Li et al., 2009; Murmu et al., 2010; Zander et al., 2011). In contrast, several of these proteins have been characterized biochemically indicating that glutaredoxins could play multiple roles, including functions as electron donors, deglutathionylation of target proteins or as Fe-S cluster sensors (Rouhier et al., 2001; Gelhaye et al., 2003; Rouhier et al., 2003, 2005; Feng et al., 2006; Vieira Dos Santos et al., 2007; Bandyopadhyay et al., 2008; Zaffagnini et al., 2008; Couturier et al., 2009b, 2011; Gao et al., 2011; Bedhomme et al., 2012).

In cyanobacteria much less is known about glutaredoxins and their functions. All cyanobacteria contain at least two different glutaredoxins: one from the classical dithiolic subgroup (class I) and one from the monothiolic subgroup (class II) (Couturier et al., 2009a). Moreover, some cyanobacteria contain genes encoding for additional dithiolic glutaredoxins (class I) or for proteins from classes V and VI. *Synechocystis* sp. PCC 6803 (hereafter *Synechocystis*) contains 3 genes coding for glutaredoxins: *ssr2061* (*grxA* also known as *grx2*) and *slr1562* (*grxB* also known as *grx1*) code for dithiolic glutaredoxins that belong to class I (Couturier et al., 2009a), while *slr1846* (*grxC* also known as *grx3*) codes for a monothiolic glutaredoxin that belongs to class II (Couturier et al., 2009a). The *Synechocystis* glutaredoxins have been implicated in responses to metals and metalloids (Lopez-Maury et al., 2009; Marteyn et al., 2009; Kim et al., 2012; Marteyn et al., 2013) and oxidative stress (Li et al., 2007; Marteyn et al., 2009). Moreover, putative targets of GrxA have been identified using a monocysteinic glutaredoxin mutant (Li et al., 2007). GrxC has been shown to be a dimer and to bind a 2Fe-2S cluster that is ligated by one cysteine from each subunit of the dimer and two molecules of GSH (Picciocchi et al., 2007; Iwema et al., 2009). In contrast, the *in vivo* functions of GrxC have not been explored in cyanobacteria.

Here we have characterized mutant strains lacking one, two, or all glutaredoxin genes, in all possible combinations, in response to several stresses in *Synechocystis*. These have showed that GrxA and GrxC play a prominent role in protecting cells from stress. Analysis of double and triple mutants showed a complex pattern of genetic interactions, suggesting that they can be involved in different pathways which are all interconnected. Furthermore, expression of *grxA* and *grxC* genes was induced by some of these stresses, while the *grxB* gene was expressed constitutively under all conditions analyzed. GrxA and GrxC proteins levels did not always correspond to their mRNA levels under these conditions suggesting that expression of glutaredoxins could be controlled also post-transcriptionally.

## Materials and methods

### Strains and culture conditions

*Synechocystis* cells were grown photoautotrophically on BG11C (Rippka et al., 1979) at 30°C under continuous illumination (50 μE·m^−2^·s^−1^) and bubbled with a stream of 1% (v/v) CO_2_ in air. For plate cultures, the medium was supplemented with 1% (wt/vol) agar. Kanamycin, chloramphenicol and spectinomycin were added to a final concentration of 50 μg mL^−1^, 20 μg mL^−1^, and 5 μg mL^−1^, respectively. BG11C medium was supplemented with different concentrations of CuSO_4_, NiSO_4_, CdCl_2_, Na_2_SeO_4_, and Na_2_SeO_3_ when indicated. Experiments were performed using cultures from the mid-logarithmic phase (3–5 μg chlorophyll mL^−1^) cultivated without antibiotics. For high light conditions, cultures were illuminated with white light at an intensity of 500 μE·m^−2^·s^−1^, and the temperature was kept at 30°C by applying a 5-cm-thick water filter. For heat shock conditions, cells were grown in a water bath at 42°C. For oxidative stress conditions, 1 mM hydrogen peroxide was added. *Synechocystis* strains and their relevant genotypes are described in Table 1.

**Table 1:** **Strains used in this work**.

**Strain**	**genotype**	**ORF mutated**	**Antibiotic resistance**	**References**
WT	*Synechocystis* sp. PCC 6803			Lab stock
SGRXA	*grxA::C.C1*	*ssr2061*	Cm	Lopez-Maury et al., 2009
SGRXB	*grxB::Sp*Ω	*slr1562*	Sp	Lopez-Maury et al., 2009
SGRXC	*grxC::C.K1*	*slr1846*	Km	Lopez-Maury et al., 2009
SGRXAB	*grxA::C.C1 grxB::Sp*Ω	*ssr2061 slr1562*	Cm Sp	Lopez-Maury et al., 2009
SGRXAC	*grxA::C.C1 grxC::C.K1*	*ssr2061 slr1846*	Cm Km	Lopez-Maury et al., 2009
SGRXBC	*grxB::Sp*Ω *grxC::C.K1*	*slr1562 slr1846*	Sp Km	Lopez-Maury et al., 2009
SGRXABC	*grxA::C.C1 grxB::Sp*Ω *grxC::C.K1*	*ssr2061, slr1562 slr1846*	Cm Sp Km	Lopez-Maury et al., 2009

### RNA isolation and northern blot analysis

Total RNA was isolated from 30 mL samples of *Synechocystis* cultures in the mid-exponential growth phase (3–5 μg chlorophyll mL^−1^). Extractions were performed by vortexing cells in the presence of phenol-chloroform and acid-washed baked glass beads (0.25–0.3 mm diameter) as previously described (Garcia-Dominguez and Florencio, 1997). Five microgram of total RNA was loaded per lane and electrophoresed in 1.2% agarose denaturing formaldehyde gels (Sambrook et al., 1989) and transferred to nylon membranes (Hybond N-Plus; Amersham). All probes were synthesized by PCR and oligonucleotide pairs used are described in Table 2 and were ^32^P-labeled with a random-primer kit (Amersham Biosciences) using α-[^32^P] dCTP (3000 Ci/mmol). Prehybridization, hybridization, and washes were in accordance with Amersham instruction manuals. All filters were stripped and re-hybridized with the constitutively expressed *rnpB* gene from *Synechocystis* as loading control (Vioque, 1992). Hybridization signals were quantified with a Cyclone Phosphor System (Packard).

**Table 2:** **Oligonucleotides used in this work**.

**Oligonucleotide**	**Sequence**
GrxA250F	GGCTGTCTCGGCAAAAATTG
GrxA250R	GGTCCAACTTGCCTGCACCATC
GrxB331F	GGCTAATTTGTTCAACTGGC
GrxB331R	CTAGGCTGGGTTAGGAGGAG
GrxC295F	GCAAGAATTGATCAGTTGGTC
GrxC295R	GCCACTTCTAACATTTCCTGC
isiAF	CATAGGTCTCGGGTGGAC
isiAR	TAAAGCTGATGGCTAATG
pgr5_F	GGAGTCACTCATATGTTCGCCC
pgr5_R	CTCAGTTTCTCGAGAATTATTG
hspA_F	CCACACATCAGGAGTTAACAT
hspA_R	TTGATCATCTAGGGTCAGGAGC

### Anti-Grx antibody production, western blotting and preparation of crude extracts from *Synechocystis* cells

Anti-GrxA and anti-GrxC antisera were obtained according to standard immunization protocols by injecting GrxA and GrxC purified proteins in rabbits. For analysis of GrxA and GrxC protein levels in *Synechocystis* cells grown under different conditions, crude extracts were prepared using glass beads in 50 mM Tris HCl pH 8.0, 50 mM NaCl. Protein concentration in cell-free extracts or purified protein preparations was determined by the method of Bradford, using ovalbumin as a standard and the specified amounts of proteins were resolved using SDS-PAGE gels. Proteins were thereafter electroblotted onto nitrocellulose membranes that were blocked in PBS containing 0.1% tween 20 and 5% of skimmed milk and incubated with anti-GrxA (1:1000) or anti-GrxC (1:3000). Anti-GSI (1:50.000) was used as loading control.

## Results

### Glutaredoxins are not essential in *Synechocystis*

**Figure 1:**
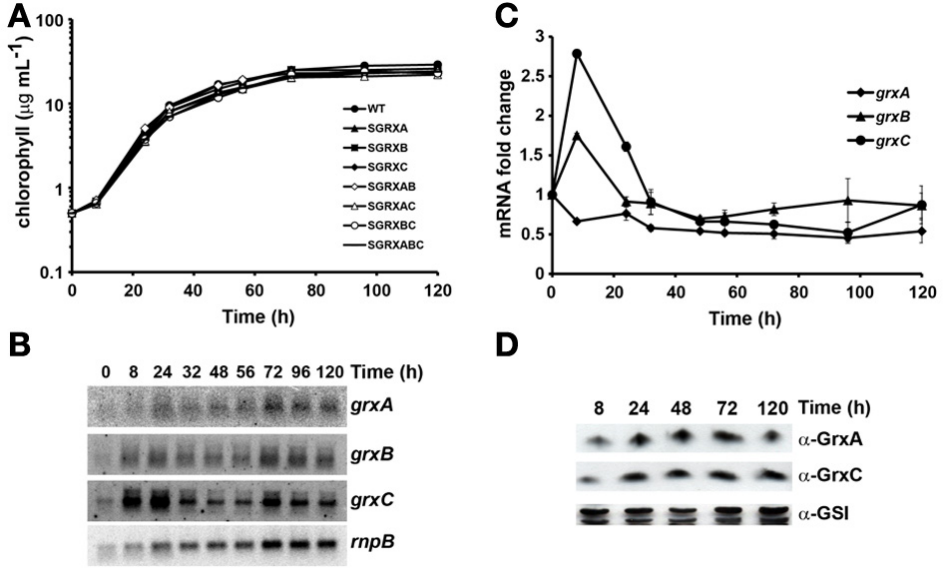
**Glutaredoxins are not essential under standard growth conditions. (A)** Semi-logarithmic representation of growth of *Synechocystis* glutaredoxin mutants strains under standard conditions. WT (•), SGRXA (▲), SGRXB (■), SGRXC (♦), SGRXAB (◊), SGRXAC (Δ), SGRXBC (○), and SGRXABC (−) strains were inoculated at 0.5 μg chlorophyll mL^−1^ and growth was monitored by measuring chlorophyll content. **(B)** Northern blot analysis of *grxA*, *grxB*, and *grxC* expression along the growth curve. Total RNA was isolated from WT cells grown in BG11C at the indicated times. The filter was hybridized with *grxA*, *grxB*, and *grxC* probes and subsequently stripped and re-hybridized with an *rnpB* probe as a control. **(C)** Quantification of relative mRNA levels of *grxA*, *grxB*, and *grxC* during the growth curve. Radioactive signals were quantified and normalized to the *rnpB* signal. Plots of relative mRNA levels vs. time were drawn; data represent average of 3 independent experiments and error bars represent SE. *grxA* (♦), *grxB* (▲), and *grxC* (•). **(D)** Western blot analysis of GrxA and GrxC levels during the growth curve. WT cells were grown in BG11C medium and cells were harvested at the indicated times. Fifteen microgram of total protein from soluble extracts were separated by 15% SDS-PAGE and subjected to western blot to detect GrxA, GrxC, and GSI.

**Figure 2:**
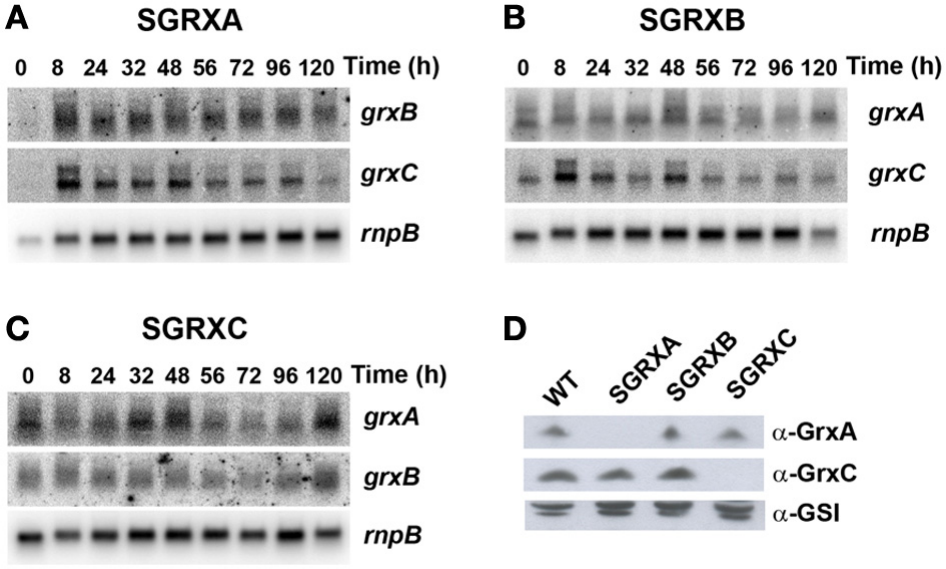
**Glutaredoxin gene expression is not altered in single glutaredoxin mutants. (A)** Northern blot analysis of *grxB* and *grxC* expression during different stages of the growth curve in SGRXA. Total RNA was isolated from SGRXA cells grown in BG11C at the indicated times. The filter was hybridized with *grxB* and *grxC* probes and subsequently stripped and re-hybridized with an *rnpB* probe as a control. **(B)** Northern blot analysis of *grxA* and *grxC* expression during different states of the growth curve in SGRXB. Total RNA was isolated from SGRXB cells grown in BG11C at the indicated times. The filter was hybridized with a *grxA* and *grxC* probes and subsequently stripped and re-hybridized with an *rnpB* probe as a control. **(C)** Northern blot analysis of *grxA* and *grxB* expression during different states of the growth curve in SGRXC. Total RNA was isolated from SGRXC cells grown in BG11C at the indicated times. The filter was hybridized with a *grxA* and *grxB* probes and subsequently stripped and re-hybridized with an *rnpB* probe as a control. **(D)** Western blot analysis of GrxA and GrxC levels in glutaredoxin mutants. WT, SGRXA, SGRXB, and SGRXC cells were grown in BG11C medium and cells were harvested at exponential phase (3–5 μg chl mL^−1^). Fifteen microgram of total protein from soluble extracts were separated by 15% SDS-PAGE and subjected to western blot to detect GrxA, GrxC, and GSI.

We have previously generated *Synechocystis* mutants lacking each or all of the glutaredoxin genes but their phenotypes were not analyzed in detail (Lopez-Maury et al., 2009). All mutant strains were viable in all possible combinations and were fully segregated when grown on solid media. Here, we have further analyzed their phenotypes in our standard growth conditions (BG11C, 30°C, 1% CO_2_ bubbled in air, 50 μE·m^−2^·s^−1^) during the growth curve. Growth of the mutants was analyzed in cultures inoculated at OD_750nm_ = 0.1 (which is equivalent to 0.5 μg chl mL^−1^) and growth was monitored following both chlorophyll content and OD_750nm_ for 5 days when it leveled off around 30 μg chl mL^−1^ (Figure 1A) and an OD_750nm_ = 6 (Figure S1). None of the strains presented a substantial reduced growth rate during the lag, exponential, linear or stationary phases of the growth curve. We have also studied expression of the glutaredoxin genes during the growth curve. Signals for all three mRNAs could be detected during all growth phases although *grxC* was induced slightly (2 to 3-fold) during the early exponential growth phase and both *grxA* and *grxB* remained almost constant during all phases (Figures 1B,C). Furthermore, we have also analyzed GrxA and GrxC protein levels during the growth phases, using specific antibodies raised against the corresponding proteins, and the proteins levels did not change substantially (Figure 1D). We have further analyzed expression of the three glutaredoxins genes during the growth curve in single mutants lacking one of the other genes in order to study if there were any compensatory effects in their expression. None of the mutant strains presented changes in mRNA levels of the remaining glutaredoxins when compared to the WT strain (Figures 2A–C, S2). We have also analyzed protein levels in exponentially growing cells (3–5 μg chl ml^−1^) of single mutant strains although neither GrxA or GrxC protein levels changed (Figure 2D). These data suggest that glutaredoxins play a less prominent role than thioredoxins under non-stress conditions and contrast with data from yeast or *E. coli* in which at least one glutaredoxin is essential for growth (Draculic et al., 2000; Fernandes and Holmgren, 2004; Ortenberg et al., 2004; Buchanan and Balmer, 2005; Toledano et al., 2013).

### Responses to different stress conditions

In order to further characterize the glutaredoxin mutant strains we have analyzed both the growth and expression of glutaredoxins in response to different stress treatments, namely high light intensity, heat shock, hydrogen peroxide stress, and metal stress.

#### High light intensity

**Figure 3:**
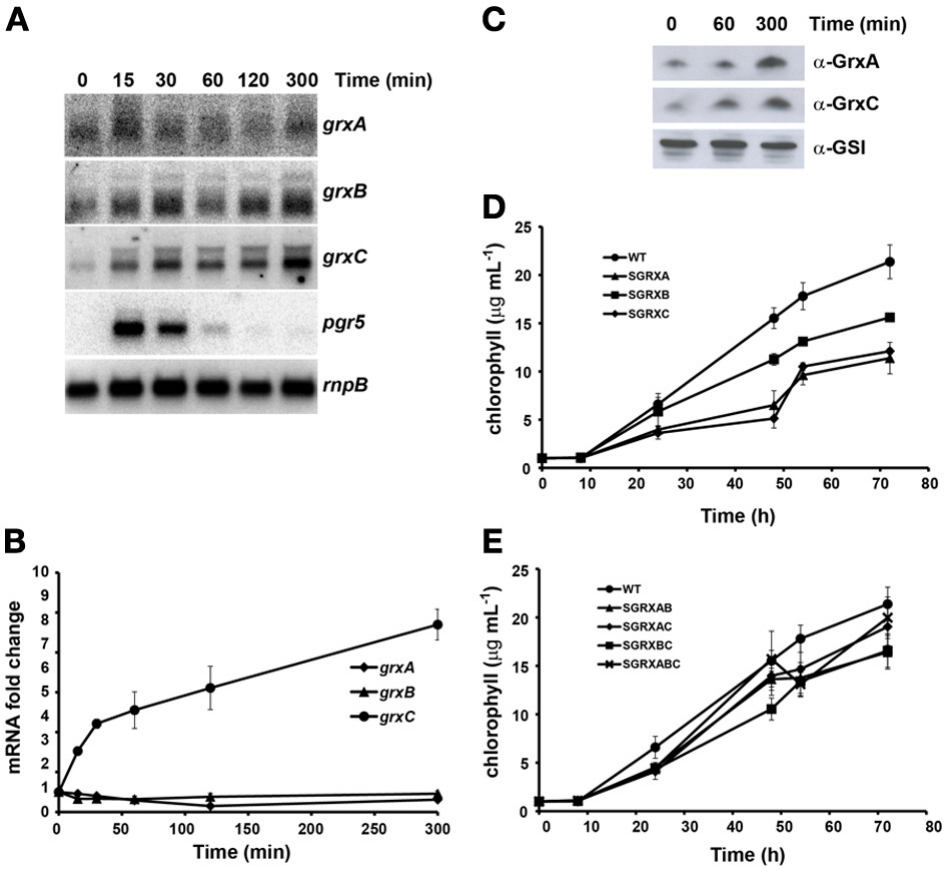
**Effect of high light on glutaredoxin gene expression and growth. (A)** Northern blot analysis of *grxA*, *grxB*, *grxC*, and *pgr5* expression in response to a shift from 50 to 500 μE·m^−2^·s^−1^ light intensity. Total RNA was isolated from exponentially growing WT cells at the indicated times after the shift. The filter was hybridized with *grxA*, *grxB*, *grxC*, and *pgr5* probes and subsequently stripped and re-hybridized with an *rnpB* probe as a control. **(B)** Quantification of relative mRNA levels of *grxA*, *grxB*, and *grxC* in response to a shift from 50 to 500 μE·m^™^·s^−1^ light intensity. Radioactive signals were quantified and normalized to the *rnpB* signal. Plots of relative mRNA levels vs. time were drawn; data represent average of 3 independent experiments and error bars represent SE. *grxA* (♦), *grxB* (▲), and *grxC* (•). **(C)** Western blot analysis of GrxA and GrxC levels in response to a shift from 50 to 500 μE·m^−2^·s^−1^. WT cells were grown in BG11C medium and samples were collected at the indicated times after the shift. 15 μg of total protein from soluble extracts were separated by 15% SDS-PAGE and subjected to western blot to detect GrxA, GrxC, and GSI. **(D)** Growth of glutaredoxin single mutants strains after a shift to high light. WT (•), SGRXA (▲), SGRXB (■), SGRXC (♦) were grown until the exponential phase, diluted to 1 μg chlorophyll mL^−1^ and shifted to high light intensity. Growth was monitored by measuring chlorophyll content. **(E)** Growth of glutaredoxin double and triple mutants strains after a shift to high light. WT (•), SGRXAB (▲), SGRXAC (♦), SGRXBC (■), and SGRXABC (x) were grown until the exponential phase, diluted to 1 μg chlorophyll mL^−1^ and shifted to high light intensity. Growth was monitored by measuring chlorophyll content.

In order to study the effect of high light treatment we transferred exponentially growing cells (3–5 μg chl mL^−1^) from our standard light intensity (50 μE·m^−2^·s^−1^) to 500 μE·m^−2^·s^−1^ and followed glutaredoxin gene expression in response to this change in light intensity. This light intensity was chosen because it did not induce photoinhibition (Figure 3) and in fact WT cells grew faster under this illumination, but was high enough to reveal a phenotype in light sensitive mutants (Perez-Perez et al., 2009a). First, we analyzed expression of glutaredoxin genes in response to this treatment; as a marker gene we have used *pgr5*, the mRNA of which has been described to be induced by high light treatment (Allakhverdiev et al., 2002). *pgr5* was transiently induced in our experiment, with a peak induction at 15 min (Figures 3A, S3A). After the shift to high light, *grxC* mRNA levels increased linearly during the 5 h of the treatment, while levels of *grxA* and *grxB* did not significantly change during the treatment (Figures 3A,B). In contrast, both GrxA and GrxC protein levels increased after this treatment (Figure 3C).

In order to clarify the roles of the three different glutaredoxins we analyzed growth of the different mutants after a shift from normal to high light. To this end, exponentially growing cells (3–5 μg chl mL^−1^) were diluted to 1 μg chl mL^−1^, shifted to 500 μE·m^−2^·s^−1^ and growth of the strains was monitored measuring chlorophyll content (Figures 3D,E) and OD_750nm_ for 72 h (Figure S3). Growth of the SGRXA (*grxA*) and the SGRXC (*grxC*) mutant strains was markedly retarded after the shift to high light while that of SGRXB (*grxB*) was only slightly reduced (Figures 3D, S3). Double and triple mutant strains behaved more similarly to SGRXB and WT strains (Figure 3E). These results suggest that both GrxA and GrxC play a role in adaptation to high light and that *grxB* mutation was able to suppress mutations in both *grxA* and *grxC*.

#### Heat shock

**Figure 4:**
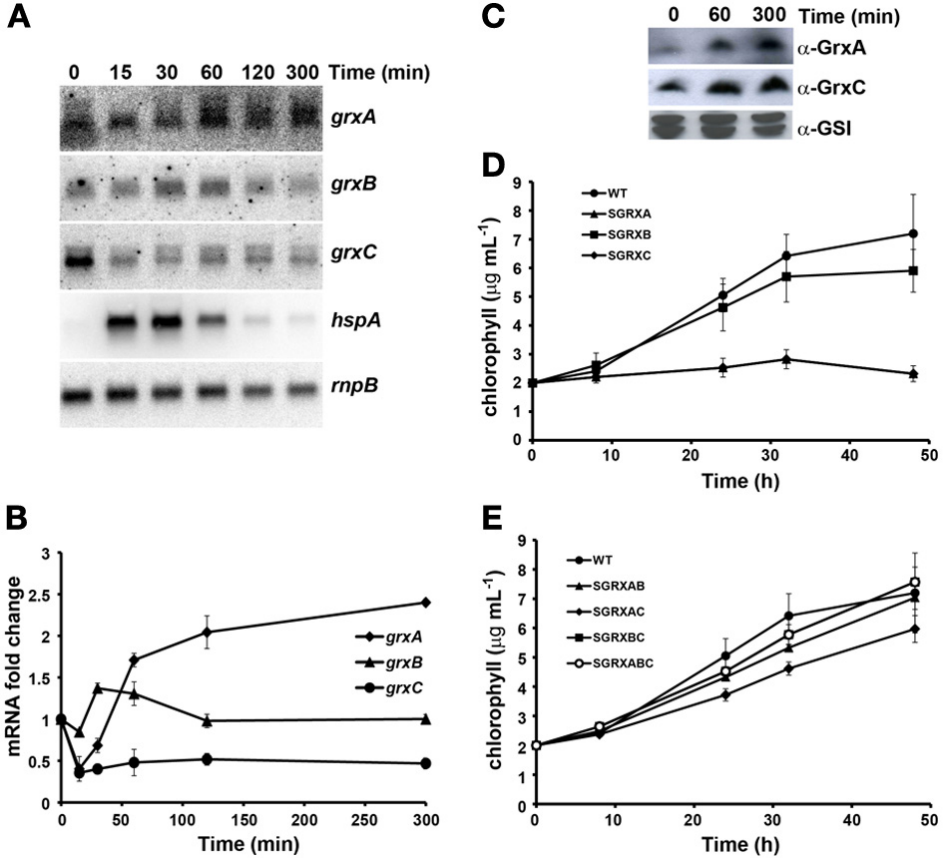
**Effect of heat shock on glutaredoxin gene expression and growth. (A)** Northern blot analysis of *grxA*, *grxB*, *grxC*, and *hspA* expression in response to heat shock. Total RNA was isolated from exponentially growing WT cells at the indicated times after the shift from 30 to 42°C. The filter was hybridized with *grxA*, *grxB*, *grxC*, and *hspA* probes and subsequently stripped and re-hybridized with an *rnpB* probe as a control. **(B)** Quantification of relative mRNA levels of *grxA*, *grxB*, and *grxC* in to response to heat shock. Radioactive signals were quantified and normalized to the *rnpB* signal. Plots of relative mRNA levels vs. time were drawn; data represent average of 3 independent experiments and error bars represent SE. *grxA* (♦), *grxB* (▲), and *grxC* (•). **(C)** Western blot analysis of GrxA and GrxC levels in response to heat shock. WT cells grown in BG11C and samples were collected at the indicated times after a shift from 30 to 42°C. Fifteen microgram of total protein from soluble extracts were separated by 15% SDS-PAGE and subjected to western blot to detect GrxA, GrxC, and GSI. **(D)** Growth of glutaredoxin single mutants strains after heat shock. WT (•), SGRXA (▲), SGRXB (■), and SGRXC (♦) were grown until the exponential phase, diluted to 2 μg chlorophyll mL^−1^ and shifted from 30 to 42°C. Growth was monitored by measuring chlorophyll content. **(E)** Growth of glutaredoxin double and triple mutant strains after heat shock. WT (•), SGRXAB (▲), SGRXAC (♦), SGRXBC (■), and SGRXABC (○) were grown until the exponential phase, diluted to 2 μg chlorophyll mL^−1^ and shifted from 30 to 42°C. Growth was monitored by measuring chlorophyll content.

We have performed experiments by shifting cells grown at 30–42°C, that is, a non-lethal heat shock treatment. As for the experiments involving high light treatment, we analyzed expression of glutaredoxins both at mRNA and protein levels. As a control we analyzed expression of the *hspA* mRNA, which was induced rapidly after the shift from 30 to 42°C and returned to almost initial levels after 5 h as previously described (Figures 4A, S4; Fang and Barnum, 2004; Suzuki et al., 2005; Tuominen et al., 2006). *grxA* mRNA levels decreased slightly during the first hour after the shift to 42°C and increased afterwards reaching more than 2 fold induction after 5 h of the treatment. In contrast *grxC* mRNA decreased 2–3 times and remained at this level along the course of the experiment, while *grxB* mRNA did not change significantly during this treatment (Figure 4B). In accordance with mRNA levels GrxA protein levels increased appreciably after 5 h (Figure 4C). However, GrxC protein levels remained constant despite the fact its mRNA levels were repressed. These results suggest that either GrxC was stabilized and/or that *grxC* mRNA translation was enhanced after heat shock.

To analyze the growth of the different strains exponentially growing cells (3–5 μg chl mL^−1^) were diluted to 2 μg chl mL^−1^, shifted from 30 to 42°C and growth of the strains was monitored measuring chlorophyll content (Figures 4D,E) and OD_750nm_ (Figures S4B,C). Analysis of mutant strains at elevated temperatures revealed that most strains showed some degree of growth inhibition. Strikingly, the SGRXA and SGRXC were completely unable to grow under this condition (Figure 4D). Surprisingly, SGRXABC, SGRXAB, SGRXBC strains, and to a lesser extent SGRXAC strain, were less sensitive to heat stress than SGRXA and SGRXC, suggesting again that mutations in *grxB* could partially suppress *grxA* and *grxC* mutations and that *grxA* and *grxC* mutation suppress partially each other. All the above results suggest that both GrxA and GrxC could play a very important role in protecting cells from heat shock.

#### Hydrogen peroxide stress

**Figure 5:**
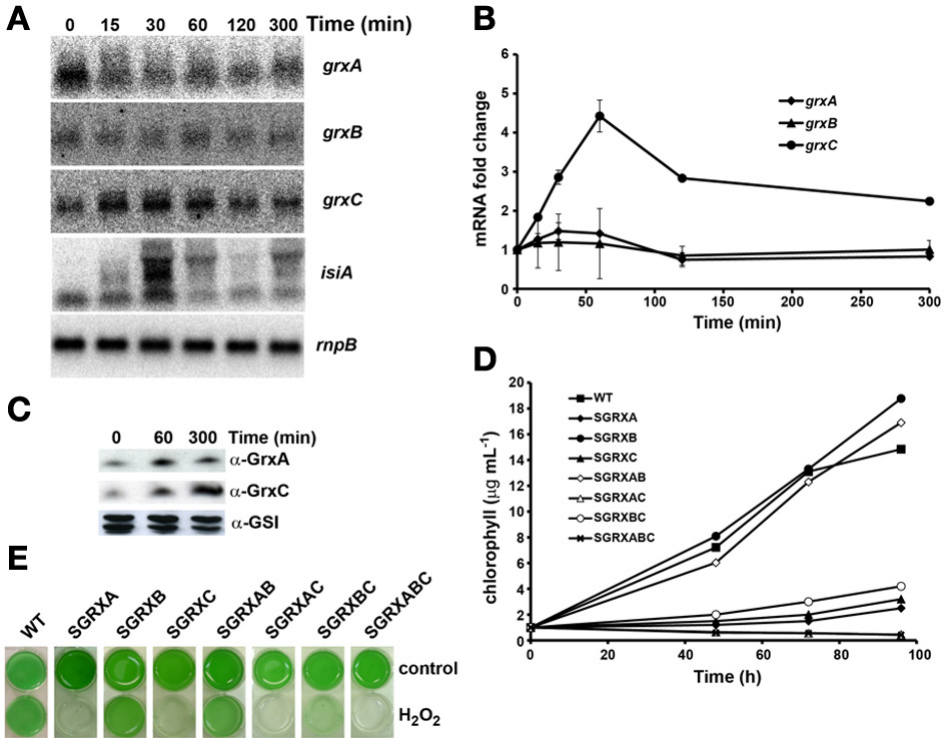
**Effect of H_2_O_2_ addition on glutaredoxin gene expression and growth. (A)** Northern blot analysis of *grxA*, *grxB*, *grxC*, and *isiA* expression in response to H_2_O_2_ addition. Total RNA was isolated from WT cells grown in BG11C at the indicated times after a 1 mM H_2_O_2_ addition. The filter was hybridized with *grxA*, *grxB*, *grxC*, and *isiA* probes and subsequently stripped and re-hybridized with an *rnpB* probe as a control. **(B)** Quantification of relative mRNA levels of *grxA*, *grxB*, and *grxC* in response to H_2_O_2_ addition. Radioactive signals were quantified and normalized to the *rnpB* signal. Plots of relative mRNA levels vs. time were drawn; data represent average of 3 independent experiments and error bars represent SE. *grxA* (♦), *grxB* (▲), and *grxC* (•). **(C)** Western blot analysis of GrxA and GrxC levels in response to hydrogen peroxide. WT cells grown in BG11C and samples were collected at the indicated times after 1mM H_2_O_2_ addition. Fifteen microgram of total protein from soluble extracts were separated by 15% SDS-PAGE and subjected to western blot to detect GrxA, GrxC, and GSI. **(D)** Growth of glutaredoxin mutants strains after H_2_O_2_ addition. WT (■), SGRXA (♦), SGRXB (•), SGRXC(▲), SGRXAB (◊), SGRXAC (Δ), SGRXBC (○), and SGRXABC (x) were grown until the exponential phase, diluted to 1 μg chlorophyll mL^−1^ and 1 mM H_2_O_2_ was added. Growth was monitored by measuring chlorophyll content. **(E)** Photograph of glutaredoxin mutants grown in liquid BG11C or BG11C supplemented with 1 mM H_2_O_2_ for 5 days.

We have analyzed the effect of addition of 1 mM of hydrogen peroxide (H_2_O_2_) both at mRNA and protein levels and on growth of the mutant strains. As a marker gene we used the *isiAB* operon, which has been reported to be induced after this treatment (Li et al., 2004a; Kanesaki et al., 2007), with a peak at 30 min in our experiments (Figures 5A, S5). *grxC* mRNA was induced transiently with a peak at 1h after the treatment and decreased afterwards. In contrast, both *grxA* and *grxB* mRNA levels remained nearly constant during the time course (Figures 5A,B). However, both GrxA and GrxC protein levels increased during the experiment with maximum levels reached after 5 h of treatment (Figure 5C). These results suggest that GrxA is stabilized in response to oxidative stress or, alternatively, that its translation is increased, while GrxC accumulation followed its mRNA induction pattern but with a delay in protein accumulation.

In order to study the sensitivity of the different strains, cells were grown until they reached the exponential growth phase (3–5 μg chl mL^−1^), then cultures were diluted to 1 μg chl mL^−1^ and 1 mM of H_2_O_2_ was added to the cultures. Growth was monitored for 4 days by measuring chlorophyll content (Figures 5D,E). Both SGRXA and SGRXC mutant strains presented a clear sensitivity to the H_2_O_2_ treatment and were unable to grow under this condition (Figures 5D,E). However, in contrast to high light and heat shock treatments, after addition of H_2_O_2_ the SGRXAC double mutant strain presented an enhanced sensitivity, suggesting that GrxA and GrxC participate in parallel pathways that cooperate in response to H_2_O_2_ (Figures 5D,E). Furthermore, mutation of *grxB* partially suppresses the sensitivity of *grxA* mutants (SGRXAB was less sensitive than SGRXA) but had almost no effect on *grxC* mutation (compare SGRXC and SGRXBC which were almost identical). The triple mutant SGRXABC showed a degree of sensitivity comparable to that of SGRXAC, which suggested that the *grxB* mutation was not able to suppress the stress sensitivity of the SGRXAC strain.

#### Metal stress

**Figure 6:**
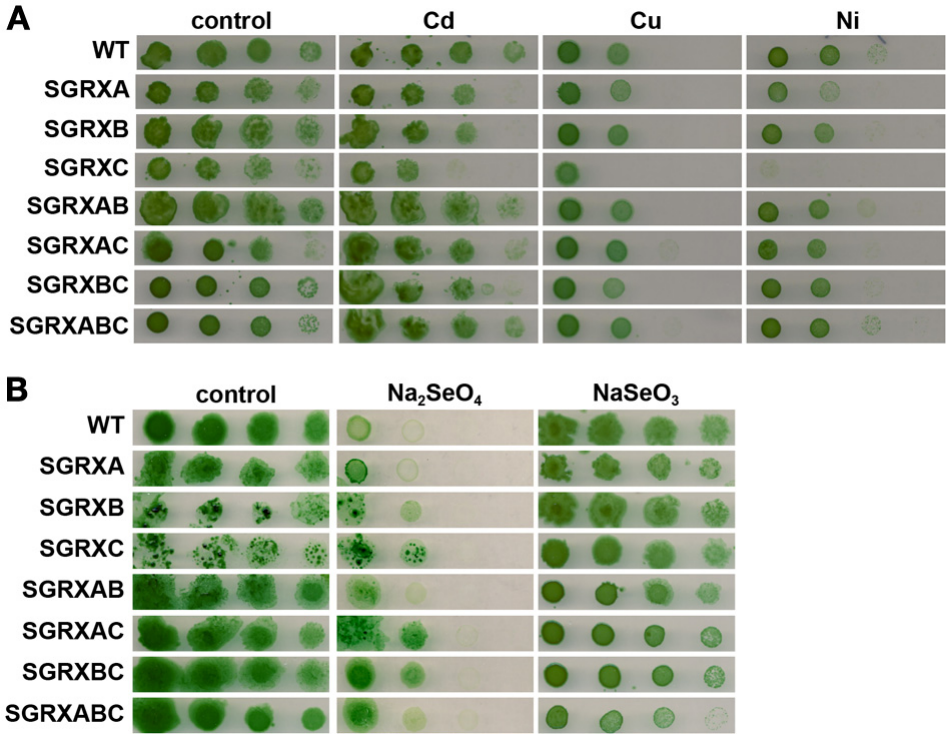
**Effect of different metals on glutaredoxin mutants growth. (A)** Sensitivity of glutaredoxin mutants to metals. Tolerance of WT, SGRXA, SGRXB, SGRXC, SGRXAB, SGRXAC, SGRXBC, and SGRXABC strains to cadmium, copper, and nickel was examined. Tenfold serial dilutions of a 1 μg chlorophyll mL^−1^ of exponentially growing cells suspension were spotted onto BG11C supplemented with 2 μM Cd, 3 μM Cu, and 5 μM Ni. Plates were photographed after 5 days of growth. **(B)** Sensitivity of glutaredoxin mutants to selenium. Tolerance of WT, SGRXA, SGRXB, SGRXC, SGRXAB, SGRXAC, SGRXBC, and SGRXABC strains to selenate (Na_2_SeO_4_) or selenite (Na_2_SeO_3_) was examined. Ten-fold serial dilutions of a 1 μg chlorophyll mL^−1^ of exponentially growing cells suspension were spotted onto BG11C supplemented with 30 μM Na_2_SeO_3_ or 30 μM Na_2_SeO_4_. Plates were photographed after 5 days of growth.

We have previously shown that GrxA was essential for arsenate resistance as mutants lacking *grxA* were sensitive to arsenate in the media and GrxA was the best electron donor *in vitro* for the main arsenate reductase from *Synechocystis* (Lopez-Maury et al., 2009). Furthermore, GrxA (Grx2) was suggested to be involved in selenate resistance (Marteyn et al., 2009) and recently GrxB has been implicated in mercury and uranium resistance (Marteyn et al., 2013). In order to further analyze the sensitivity of glutaredoxin mutants to different metals, we have analyzed the growth of all mutants in the presence of selenate, selenite, cadmium, copper, and nickel. All mutant strains, except SGRXC, grew as well as the WT in the presence of 2 μM of Cd, 3 μM of Cu, 5 μM of Ni and 30 μM Na_2_SeO_3_ or 30 μM Na_2_SeO_4_ (Figure 6). The SGRXC strain presented hypersensitivity to nickel and copper, and to a lesser extent to Cd (Figure 6A), while the double mutants SGRXAC and SGRXBC (lacking *grxA* or *grxB* in addition to *grxC*) and triple mutants (lacking all glutaredoxin genes) strains behaved like the WT. This suggests that compensatory mechanisms were activated in double and triple mutant strains that protect against the toxic effect of these metals. However, although these mutations were able to suppress *grxC* mutation, they did not enhance resistance in a WT background (Figure 6A).

## Discussion

Here we have extensively characterized the complete set of glutaredoxins in the cyanobacterium *Synechocystis*, a model photosynthetic prokaryote. The characterization of the different glutaredoxin mutant strains have revealed that none of the glutaredoxin genes are required under normal growth conditions but that they play an important role in protecting cells from environmental stresses. Our data also indicated that there are complex genetic interactions between glutaredoxins genes and that these interactions are stress dependent. The fact that we have not observed any compensatory effects on expression of glutaredoxin genes or GrxA and GrxC protein levels in mutants lacking other glutaredoxin genes, would suggest that the phenotypes we have observed are most probably linked to downstream processes regulated by glutaredoxins and not to dosage-dependent effects. Furthermore, analysis of the expression of the glutaredoxin genes has shown that these genes were not all under the same regulatory system because their patterns of expression were not coordinated. In fact, the *grxB* mRNA level did not change significantly under any of the conditions tested, while *grxA* was only induced by heat shock and *grxC* was induced after H_2_O_2_ and during high light treatment but repressed by heat shock. Although *grxC* is induced by high light intensities and H_2_O_2_, this gene is not under the control of any of the regulatory proteins described to respond to these stress conditions in *Synechocystis* (Hsiao et al., 2004; Kobayashi et al., 2004; Suzuki et al., 2005; Jantaro et al., 2006; Tuominen et al., 2006; Kanesaki et al., 2007; Tuominen et al., 2008; Horiuchi et al., 2010; Singh et al., 2010; Muramatsu and Hihara, 2012). On the other hand, GrxA protein levels increased under all stress conditions, while the GrxC protein levels were induced only after high light and H_2_O_2_, following the changes of its mRNA levels, and was maintained constant after heat shock, despite repression of its mRNA levels. This suggests that in addition to a transcriptional regulation there are also other levels of regulation at the protein level for both glutaredoxins.

Analysis of growth the mutant strains showed that two of the glutaredoxins, GrxA and GrxC, play a prominent role in cell protection against stress, as the single mutants lacking *grxA* or *grxC* were sensitive to several stresses. Both GrxA and GrxC were necessary for oxidative stress defense, since mutants lacking one of them were extremely sensitive to the presence of external H_2_O_2_ and the mutant lacking both genes showed and additive phenotype (Figure 5). The single mutant lacking *grxA* (the SGRXA strain) proved to be hypersensitive to H_2_O_2_, which is in agreement with phenotypes previously described for this mutant (Li et al., 2007; Marteyn et al., 2009), but the mechanism for this sensitivity and the targets of GrxA are still unknown. GrxA is able to decompose H_2_O_2_ *in vitro* although it is much less efficient than most peroxiredoxins, which are in fact reduced by thioredoxins and not by glutaredoxins in *Synechocystis* (Perez-Perez et al., 2009b). Therefore, it is unlikely that this activity could account for the protection of cells against H_2_O_2_. Besides that, GrxA has been shown to interact with both catalase and peroxiredoxin II (PrxII), although the catalase activity was inhibited by GrxA in *Synechocystis* extracts (Li et al., 2007) and PrxII eventually did not accept electrons from GrxA (Perez-Perez et al., 2009b). However, it can not be ruled out that glutathionylation is needed for activation and/or protection of these two proteins under oxidative stress conditions. The SGRXA strain was also sensitive to high light and GrxA protein levels increased after this treatment suggesting that GrxA could play a role in high light acclimation. During the high light treatment the excess of light absorbed can generate reactive oxygen species (ROS) leading to oxidative damage and this ROS might affect the growth of the SGRXA strain. Furthermore, the SGRXA strain was also extremely sensitive to heat shock which suggests that GrxA could also play an important role during heat stress. In this regard both GroEL and DnaK1 (two chaperones that are essential for heat adaptation) were also identified as GrxA targets (Li et al., 2007), which suggests that GrxA could be needed for activation or as a helper of both chaperones to function in response to heat shock. Similar phenotypes with respect to oxidative stress sensitivity and heat shock sensitivity have been reported for glutaredoxin mutants in other bacteria (Prinz et al., 1997; Fernandes and Holmgren, 2004; Li et al., 2004b; Benyamina et al., 2013) and in yeast (Luikenhuis et al., 1998; Draculic et al., 2000; Chung et al., 2004), suggesting that dithiolic glutaredoxins could have conserved roles in microorganisms. Finally, we have tested whether SGRXA strains were sensitive to metals and selenium compounds. Any of the tested metals affected significantly the growth of *grxA* mutant strains (Figure 6), including selenate or selenite which is in disagreement with previous data (Marteyn et al., 2009). Sensitivity to metals is highly influenced by growth conditions, especially light conditions, and this could explain the differences in selenium sensitivity between our results and previously published ones (Marteyn et al., 2009).

The SGRXC strain is unique in that it showed hypersensitivity to all stresses tested. GrxC is a prototypical monothiolic glutaredoxin which has been shown to contain an oxygen labile 2Fe-2S cluster (Picciocchi et al., 2007), and the *grxC* gene is able to complement the defects of a *grx5* yeast mutant (Molina-Navarro et al., 2006). In several organisms it is well-documented that monothiolic glutaredoxins impact Fe-S cluster synthesis and/or assembly although their specific roles are still under discussion. Yeast Grx5 is involved in mitochondrial Fe-S cluster assembly and mutants lacking this protein showed symptoms of oxidative stress under standard growth conditions, accumulation of apoproteins lacking Fe-S clusters in the mitochondria and were unable to grow under aerobic conditions (Rodriguez-Manzaneque et al., 1999, 2002; Belli et al., 2004). A similar phenotype has also been described for a monothiolic glutaredoxin mutant in *Sinorhizobium meliloti* (Benyamina et al., 2013). Furthermore, in yeast it has been shown that GSH is essential for Fe-S cluster assembly but in this case it affects both cytoplasmic and mitochondrial Fe-S clusters, suggesting a more global impact in Fe-S cluster assembly. This is probably related to its role in binding Fe-S clusters in all monothiolic glutaredoxins in yeast cells (Kumar et al., 2011). In photosynthetic organisms the *in vivo* role of monothiolic glutaredoxins has been analyzed only in *Arabidopsis* which has four class II monothiolic glutaredoxins (AtGrxS14–17; Couturier et al., 2009a). Of these AtGrxS14 and AtGrxS16 have been localized to the chloroplast, AtGrxS15 is localized in the mitochondria and chloroplast and AtGrxS17 is a cytosolic protein (Cheng et al., 2006; Bandyopadhyay et al., 2008; Cheng, 2008). It was also shown that both AtGrxS14 and AtGrxS15 mutants are hypersensitive to H_2_O_2_ (Cheng et al., 2006; Cheng, 2008), while the AtGrxS17 mutant is sensitive to heat shock and has higher ROS contents under this condition (Cheng et al., 2011). These proteins also present a labile 2Fe-2S cluster (like GrxC) which *in vitro* could be transferred to *Synechocystis* ferredoxin (Bandyopadhyay et al., 2008; Liu et al., 2013). Furthermore, these proteins are also able to complement the yeast *grx5* mutant strain, like *grxC*, suggesting that they are functionally conserved (Bandyopadhyay et al., 2008; Liu et al., 2013). Recently it has been shown that the *E. coli* monothiolic glutaredoxin (GrxD) is able to transfer its Fe-S cluster to ferredoxin (Yeung et al., 2011) but not to MiaB, a radical AdoMet enzyme containing two 4Fe-4S clusters, despite holoGrxD being able to interact with MiaB. This led these authors to propose that GrxD is not involved in Fe-S cluster assembly but in Fe-S cluster repair (Boutigny et al., 2013). This role in repair and/or synthesis of Fe-S cluster could explain SGRXC sensitivity to different stresses, because Fe-S clusters are vulnerable to many stresses, especially H_2_O_2_ and metal stress (Imlay, 2006, 2013). In fact destabilization of Fe-S clusters in essential enzymes constitute the main mechanism for metal toxicity in several organisms, including *Synechocystis*, suggesting that this is a common problem when cells are challenged with an excess of metals (Ranquet et al., 2007; Macomber and Imlay, 2009; Chillappagari et al., 2010; Fantino et al., 2010; Tottey et al., 2012; Xu and Imlay, 2012). The target proteins which require GrxC for Fe-S cluster assembly and/or repair in *Synechocystis* remain unidentified, but because the SGRXC strain is viable and did not show any growth defects under non-stressed conditions, its targets are unlikely to be essential. Alternatively GrxC could be involved only in Fe-S cluster repair under stress conditions. This is further supported by GrxC induction after several stress treatments, suggesting that cellular requirements for GrxC under these conditions may increase.

In contrast the SGRXB strain did not display any obvious phenotype, neither were the *grxB* mRNA levels altered under any of the conditions tested, suggesting that this protein has a minor role and/or a very specialized role. This is supported by the recent data, which showed that *grxB* mutants were sensitive to mercury and uranium. GrxB regulates the activity of MerA, a mercury reductase, via glutathionylation of one of its active cysteines (Marteyn et al., 2013) and mutation of both *merA* or *grxB* confers sensitivity to these compounds. Surprisingly, our results showed that *grxB* mutation was able to partially suppress most of the phenotypes of *grxA* mutant strains suggesting that it could be negatively controlling functions activated by GrxA. This is further supported by data showing physical interaction between GrxA and GrxB, which has been observed by two different groups using different experimental approaches (Li et al., 2007; Marteyn et al., 2009). This suggests that these interactions are *bona fide* interactions and that these proteins could regulate each other's activities. Furthermore, GrxA and GrxB have been proposed to be reduced by *Synechocystis* NADPH-thioredoxin reductase (encoded by *slr0600;* NTR), which present homology to bacterial NADPH thioredoxin reductases. Which redoxins are reduced by NTR is controversial because both dithiolic glutaredoxins, GrxA and GrxB, and TrxA have been reported to be reduced by NTR (Li et al., 2007; Hishiya et al., 2008; Marteyn et al., 2009). In addition, a *Synechocystis* mutant lacking GSH also presented stress sensitivity, although its phenotypes were more severe (Cameron and Pakrasi, 2011) than phenotypes of glutaredoxin mutants described here. This suggests that, as described in yeast (Kumar et al., 2011), GSH could have additional roles in *Synechocystis* which are not mediated solely by glutaredoxins. One possibility is that the NTR-Grx1/2 or the NTR-TrxA system could work as alternative pathways to reduce GSH in *Synechocystis*, because this strain lacks a gene for a canonical glutathione reductase (GR) and NADPH dependent GR activity is not detected in extracts (Marteyn et al., 2009; Cameron and Pakrasi, 2011). Similar results have been obtained in yeast in which mutant lacking GR can use both Grx2 and Trx2 to reduce GSH (Morgan et al., 2013). All the above data suggest that there could be a cross-regulation between the thioredoxin and glutaredoxin systems in *Synechocystis* as it has been shown in other organisms including plants and algae (Gelhaye et al., 2003; Michelet et al., 2005; Rouhier et al., 2008; Zaffagnini et al., 2008; Meyer et al., 2009, 2012; Lillig and Berndt, 2013; Toledano et al., 2013). Finally, analysis of double mutants with *grxC* shows that *grxB* was also able to suppress totally or partially the sensitivity phenotype of *grxC* single mutants to high light, heat shock and metal stress but not to oxidative stress. The relation between these two proteins is completely unknown but is possible that *grxB* affects GSH metabolism which will probable also alter GrxC function.

In conclusion, we have shown that glutaredoxins are essential for adaptation to several stresses in *Synechocystis* and that GrxA and GrxC levels increase in response to stress treatments. This induction is mediated mainly by transcriptional induction for GrxC and by posttranscriptional mechanisms in the case of GrxA. Analysis of the different genetic interactions between the glutaredoxin genes suggest the existence of an interconnected genetic network that is perturbed in response to environmental changes. In this regard, it would be interesting to analyze mutants lacking both glutaredoxins and other components of antioxidative system, such as the thioredoxins, as some of the phenotypes we have observed are shared between them.

## Conflict of interest statement

The authors declare that the research was conducted in the absence of any commercial or financial relationships that could be construed as a potential conflict of interest.
