# Supplementary material for: Glutaredoxins are essential for stress adaptation in the cyanobacterium Synechocystis sp. PCC 6803
Source: Front Plant Sci. 2013 Nov 4;4:428. doi: 10.3389/fpls.2013.00428 (PMC3816324; doi:10.3389/fpls.2013.00428)
Supplement: Supplementary file 1 [file Presentation1.PDF]

Figure S1

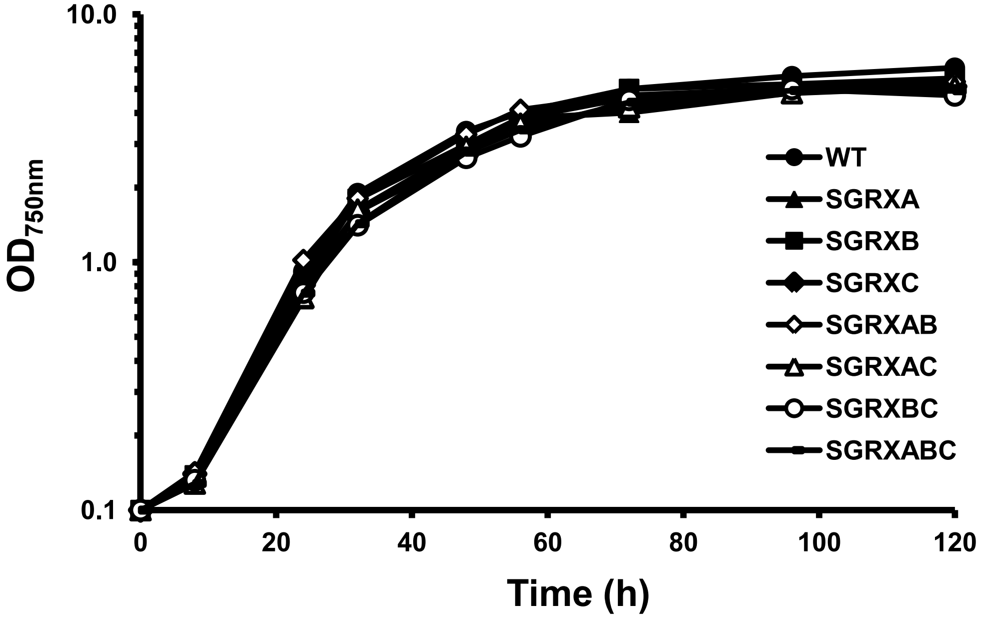

Figure S1. Glutaredoxins are not essential under standard growth conditions. Semi-logarithmic representation of growth of *Synechocystis* glutaredoxin mutants strains under standard conditions. WT (●), SGRXA (▲), SGRXB (■), SGRXC(♦), SGRXAB (◊), SGRXAC (Δ), SGRXBC (○) and SGRXABC (-) strains were inoculated at 0.1 OD_{750nm} and growth was monitored by measuring OD_{750nm} .

Figure S2

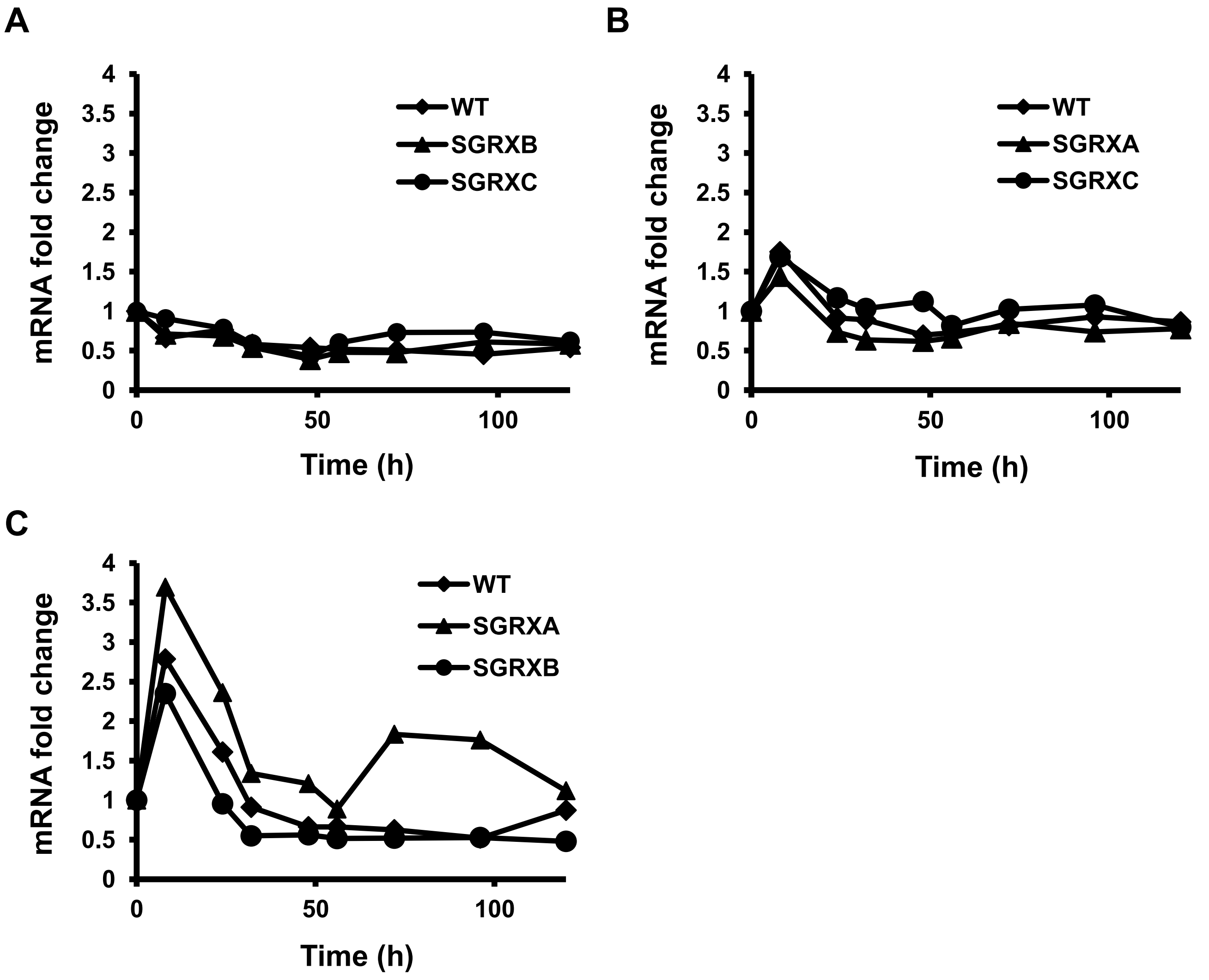

Figure S2. Glutaredoxins gene expression is not altered in single glutaredoxin mutants.

A. Quantification of relative mRNA levels of *grxA* during the growth curve in WT, SGRXB and SGRXC. Radioactive signals were quantified and normalized to the *rnpB* signal. Plots of relative mRNA levels versus time were drawn; data represent average of 3 independent experiments for the WT strain and 2 for mutants strains. WT (♦), SGRXB (▲) and SGRXC (●).
B. Quantification of relative mRNA levels of *grxB* during the growth curve in WT, SGRXB and SGRXC. Radioactive signals were quantified and normalized to the *rnpB* signal. Plots of relative mRNA levels versus time were drawn; data represent average of 3 independent experiments for the WT strain and 2 for mutants strains. WT (♦), SGRXA (▲) and SGRXC (●).
C. Quantification of relative mRNA levels of *grxC* during the growth curve in WT, SGRXA and SGRXB. Radioactive signals were quantified and normalized to the *rnpB* signal. Plots of relative mRNA levels versus time were drawn; data represent average of 3 independent experiments for the WT strain and 2 for mutants strains. WT (♦), SGRXA (▲) and SGRXB (●).

Figure S3

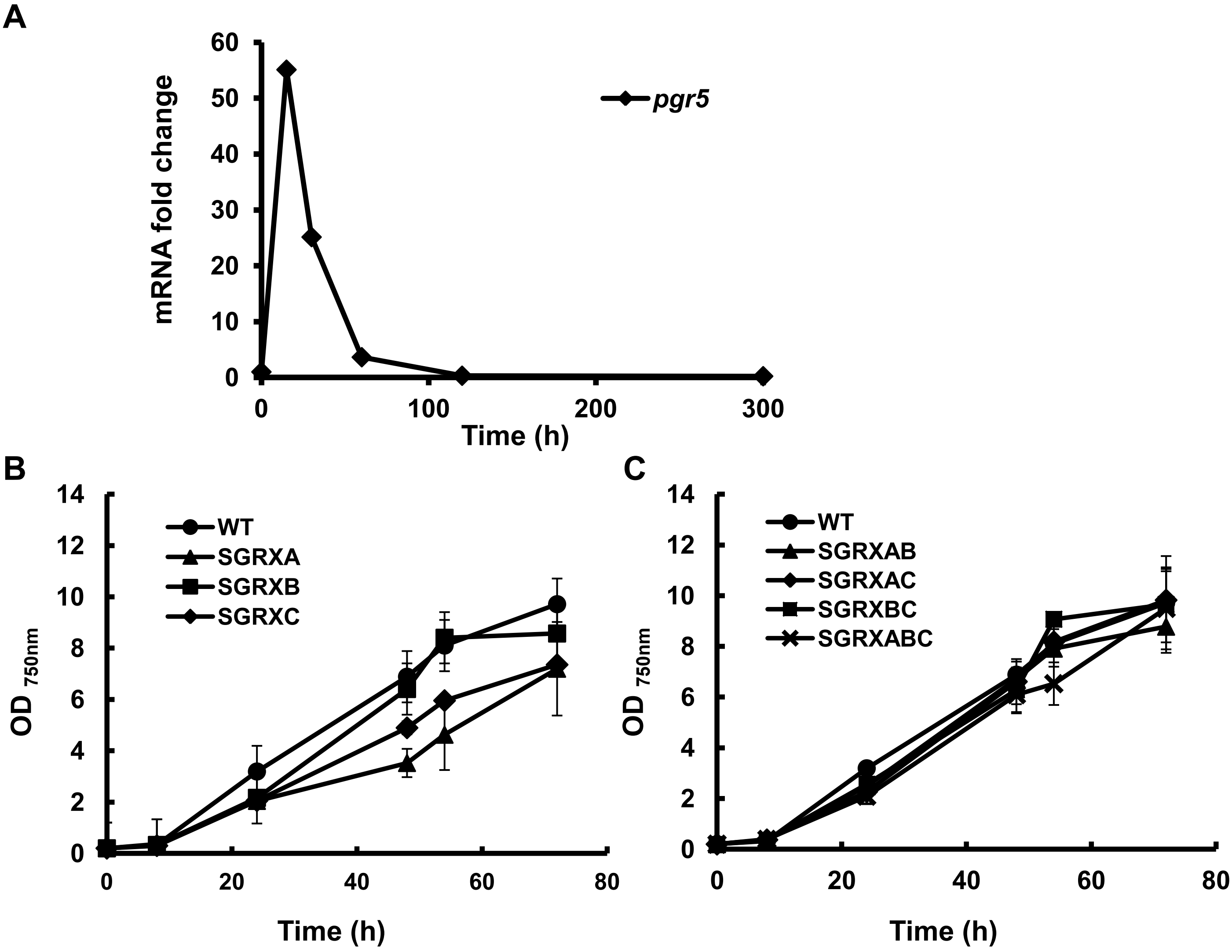

Figure S3. Effect of high light on gene expression and growth of glutaredoxin mutants.

A. Quantification of relative mRNA levels of *pgr5* in response to a shift from 50 to 500 μE m^{-2} s^{-1} light intensity. Radioactive signals were quantified and normalized to the *rnpB* signal. Plots of relative mRNA levels versus time were drawn; data represent average of 2 independent experiments with similar results.

B. Growth of glutaredoxin single mutants strains after a shift to high light. WT, SGRXA , SGRXB and SGRXC were grown until the exponential phase, diluted to 0.2 OD_{750nm} and shifted to from 50 to 500 μE m^{-2} s^{-1}. Growth was monitored by measuring OD_{750nm}.

C. Growth of glutaredoxin double and triple mutants strains after a shift to high light. WT , SGRXAB , SGRXAC , SGRXBC and SGRXABC were grown until the exponential phase, diluted to 0.2 OD_{750nm} and shifted to from 50 to 500 μE m^{-2} s^{-1}. Growth was monitored by measuring OD_{750nm}.

Figure S4

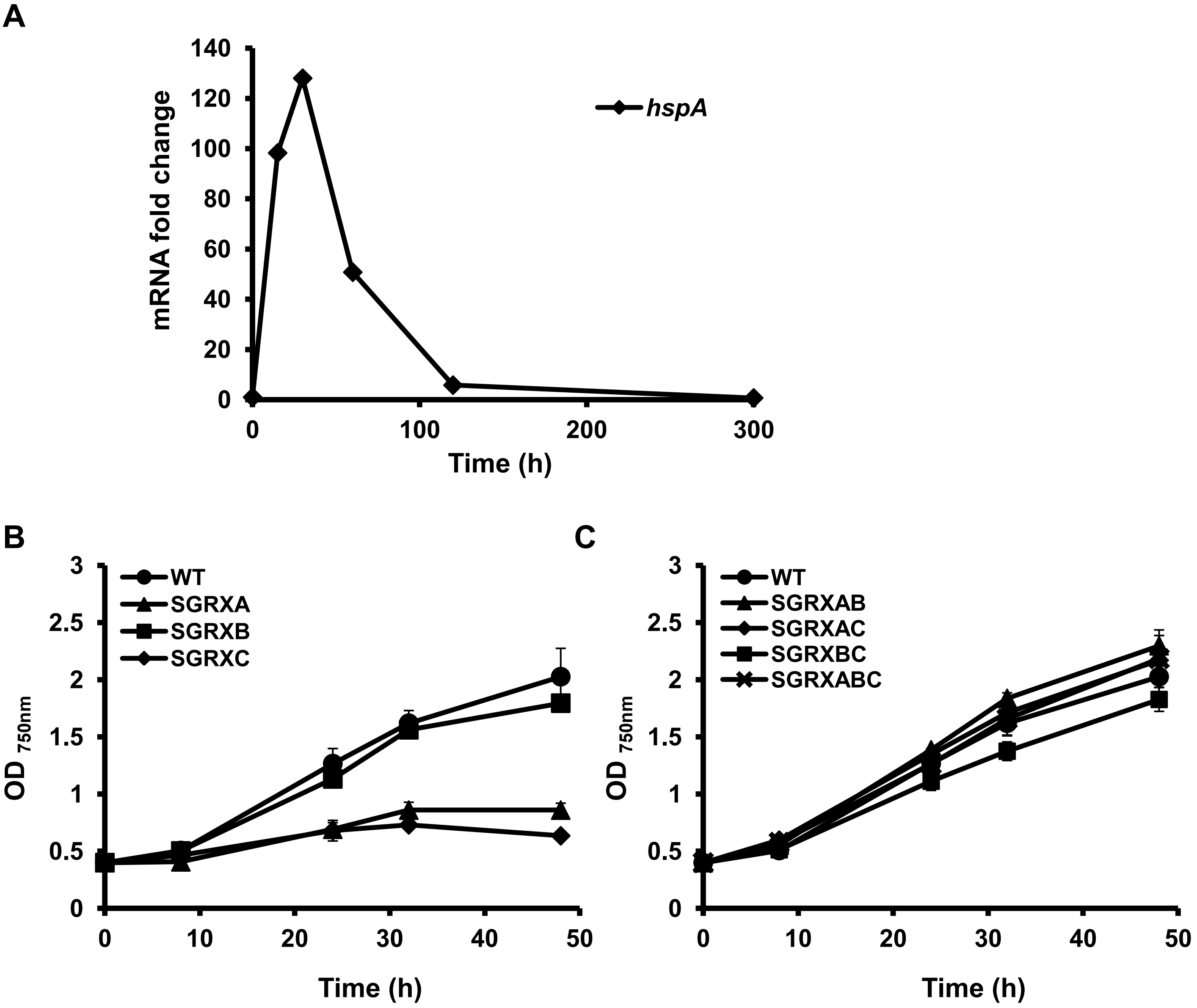

Figure S4. Effect of heat shock on gene expression and growth of glutaredoxin mutants.

A. Quantification of relative mRNA levels of expression in response to heat shock. Radioactive signals were quantified and normalized to the *rnpB* signal. Plots of relative mRNA levels versus time were drawn; data represent average of 2 independent experiments with similar results.

B. Growth of glutaredoxin single mutants strains after a shift to high light. WT, SGRXA , SGRXB and SGRXC were grown until the exponential phase, diluted to 0.4 OD_{750nm} and shifted to 42 ºC. Growth was monitored by measuring OD_{750nm}.

C. Growth of glutaredoxin double and triple mutants strains after a shift to high light. WT , SGRXAB , SGRXAC , SGRXBC and SGRXABC were grown until the exponential phase, diluted to 0.4 OD_{750nm} and shifted to 42 ºC. Growth was monitored by measuring OD_{750nm}.

Figure S5

A

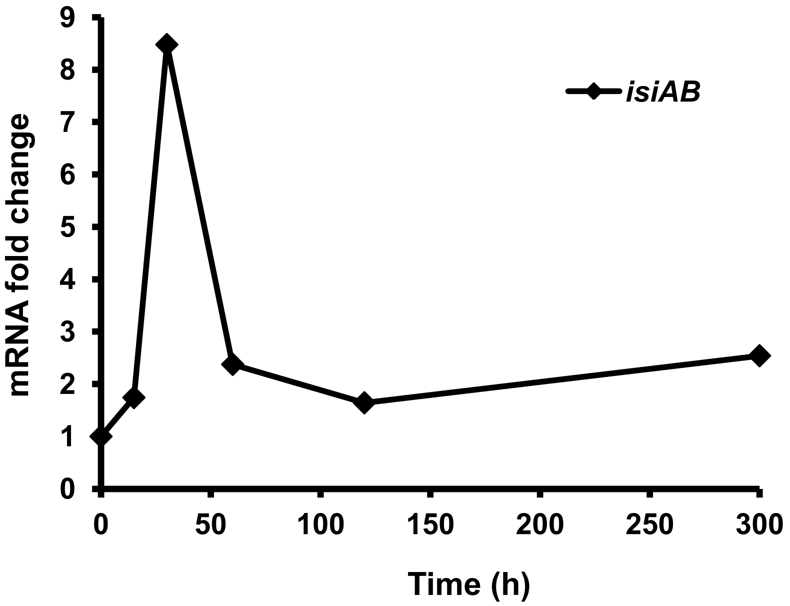

Figure S5. Effect of H_2O_2 addition on *isiAB* gene expression.
Quantification of relative mRNA levels of *isiAB* in response to to H_2O_2 addition. RNA levels were normalized with the *rnpB* signal. Plots of relative mRNA levels versus time were drawn; data represent average of 2 independent experiments with similar results.
